# Zinc Oxide Nanoparticles Functionalized on Hydrogel Grafted Silk Fibroin Fabrics as Efficient Composite Dressing

**DOI:** 10.3390/biom10050710

**Published:** 2020-05-04

**Authors:** Sudip Majumder, Ujjwal Ranjan Dahiya, Sunny Yadav, Pratibha Sharma, Debashree Ghosh, Gyandshwar K. Rao, Varun Rawat, Gaurav Kumar, Anuj Kumar, Chandra Mohan Srivastava

**Affiliations:** 1 Department of Chemistry, Amity School of Applied Sciences, Amity University Haryana, Gurugram 122413, India; sudip22m@gmail.com (S.M.); sunnyyadavmed121296@gmail.com (S.Y.); pratibha30sharmas@gmail.com (P.S.); dghosh@ggn.amity.edu (D.G.); gkrao@ggn.amity.edu (G.K.R.); vrawat@ggn.amity.edu (V.R.); 2 CSIR-Institute of Genomics and Integrative Biology, New Delhi 110021, India; ujjwal.ranjan@igib.in; 3 Department of Biochemistry, University of Delhi, South Campus, New Delhi 110021, India; gauravkumar747@gmail.com; 4 School of Chemical Engineering, Yyeongsan 38541, Korea; 5 Centre for Polymer Technology, Amity School of Applied Sciences, Amity University Haryana, Gurugram 122413, India

**Keywords:** silk fibroin, zinc oxide nanoparticles, hydrogel, antimicrobial activity, cytocompatibility

## Abstract

Recent advances in woundcare is targeted towards developing active-dressings, where multiple components are combined to provide a suitable environment for rapid healing. The aim of the present research is to study the preparation of biomimic composite wound dressings by the grafting of hydrogel on silk fibroin fabric. The swelling ability of hydrogel grafted silk fibroin fabric was optimized by changing the initiator concentration. In order to impart antimicrobial properties, these dressing are further coated sono-chemically with zinc oxide nanoparticles. The water vapor transmission rate of the prepared samples was measured. The conformation of silk fibroin proteins after grafting with hydrogel was also confirmed using Fourier Transform Infrared Spectroscopy (FTIR). The morphology of the zinc oxide-coated silk fibroin fabric and hydrogel-coated silk fibroin was studied using Scanning Electron Microscope (SEM). The antimicrobial activity of the zinc oxide-coated samples was studied against *E coli*. The cytocompatibility of the prepared dressing materials were evaluated using L929 fibroblast cells. MTT assay and phase contrast microscopic studies showed that the adherence, growth, and proliferation of the L929 fibroblast cells that were seeded on zinc oxide nanoparticles on the functionalized hydrogel-coated silk fibroin dressing was significantly higher than that of pure silk fibroin due to the highly porous, bio-mimic structure that allowed ease of passage of nutrients, growth factors, metabolites, and the exchange of gases which is beneficial for successful regeneration of damaged tissues. The expression of TNF-α and IL-2 were not significantly higher than that of control. The proposed composite dressing would be a promising material for wound dressing and regenerative medicine but in order to prove the efficacy of these materials, more in vivo experiments and clinical tests are required to be conducted in future.

## 1. Introduction

Any disruption to the living tissue in which the affected part is torn away or punctured is defined as a wound [1]. Wounds can be categorized as acute or chronic [2] based on the duration of its cure. Treatment and cure of these wounds require proper wound dressing which plays a significant role as a therapeutic agent in keeping the wound safe from further infection. The dressing acts as a barrier against the penetration of bacteria to the wound environment. There exists a requirement for a suitable material that would cover the wound to prevent it from infection for effective wound healing [3]. Various materials such as hydrogel, aerogel, xerogel, microfiber, nanofiber, non-woven fabric, and gels as well as the autograft, allograft, and xenograft have been used to deliver functional molecules to the wound site. However, these graft procedures are associated with several limitations. For example, there is limited availability of cells in the case of severely burnt patients and there may rejection and infection of the dressing allograft. Thus, there is a need for developing dressings that can provide tissue compatibility, high mechanical and tensile strength, and low bacterial infection.

Hydrogels are three dimensional crosslinked networks of the water-insoluble polymer [4] which have desirable characteristics of an ideal dressing. The main limitations with the swell hydrogel is their poor mechanical strength [5], non-natural adherence to the skin [6], and their limited ability to diffuse from the target site. The dry hydrogel is too hard and can cause mechanical abrasion to the wound site. To overcome this issue, a secondary layer can be introduced to fasten them in a position [7,8]. But these types of dressings also have some limitations like low water vapor transmission and flexibility. To address this issue, we prepared composite dressing materials in which Tasar silk fibroin fabric was used as a secondary dressing material, which was graft coated with hydrogel [9,10]. The Tasar variety of silk is extremely useful for biomedical applications as it contains tripeptide sequence R(Arg)-G(Gly)-D(Asp). Positively charged argentine residues attract the negatively charged cells which improves the adherence, growth, and proliferation of seeded cells [11]. These hydrogel grafted dressing materials show very high porosity, water vapor transmission, swelling ability, and structurally mimics the natural extracellular matrices [12,13]. Zinc oxide (ZnO) nanoparticles are well-studied and characterized antimicrobial agents in literature, and show biocompatibility to mammalian cells [14]. In-fact many ZnO nanoparticle-based formulations are available as a commercial product for photoprotection [15]. For this study ZnO nanoparticles of a mean size 29 nm were prepared and used. In order to introduce the antimicrobial property [16], ZnO nanoparticles (NPs) were uniformly deposited on the surface of the hydrogel-grafted Tasar silk fibroin dressing by high-intensity ultrasound. The physical, mechanical, morphological, cytocompatibility, and cytotoxic properties of the ZnO oxide nanoparticle-coated hydrogel grafted on the silk fibroin dressing were studied in order to check their efficacy for wound dressing application.

## 2. Experimental Section

### 2.1. Materials

Analytical grade chemicals were used in the present study. Reagent grade acrylamide (AAm) and *N*,*N*’-bismethylacrylamide (MBA) were brought from Spectrochem Pvt. Ltd. (Mumbai, India). Acrylic acid (AAc) and ammonium persulphate (APS) were purchased from Central Drug House Pvt. Ltd. (CDH, New Delhi, India) and were used as received. Zinc acetate, sodium hydroxide, and ethanol were purchased from CDH. All the chemicals were used as received without further purification. Double distilled water was used throughout the experiment having conductivity of 0.05 µS/cm.

### 2.2. Preparation of Composite Wound Dressing Material

Silk woven fabric (thickness ~0.20 mm) was cut into pieces (10 cm × 10 cm). These pieces were immersed in a solution of ammonium persulphate (APS) (25% *w*/*v*) for 24 h. The samples were thoroughly washed with distilled water and squeezed between two filter papers to remove any excess solution [17].

### 2.3. Hydrogel Grafting on Tasar Silk Fibroin Fabric

The APS-treated silk woven fabric was first grafted with acrylamide (7.5 g) and acrylic acid (7.5 mL) in 100 mL water. Grafting of poly(acrylamide-co-acrylic acid) on silk, *N*,*N*’-methylene bisacrylamide (MBA) (5% wt/wt of the monomer) was added. The grafting and crosslinking reaction were carried out in a beaker for 45 min at 60 °C. Samples were washed with water to extract homopolymer as well as unreacted monomers. The grafted samples were dried under vacuum until a constant weight was obtained [17].

### 2.4. Synthesis of Zinc Oxide NPs

An aqueous solution of zinc acetate (20 mL, 1 M) and sodium hydroxide (10 mL, 2M) was prepared. The sodium hydroxide solution was slowly added into the solution of zinc acetate with continuous stirring. The solution becomes viscous with the addition of sodium hydroxide and -then cleared [13]. Ethanol was added to this solution until the precipitation was completed. The solution was centrifuged at 5000 rpm and the resulting solid was washed with distilled water several times.

### 2.5. Sonochemical Coating of Dressing Material with ZnO

Zinc oxide (50 mg) NPs were dispersed in 100 mL distilled water. Hydrogel coated silk woven fabric was placed into this solution and sonicated for 30 min in a bath sonicator (5–6 times) for uniform coating of ZnO nanoparticles on the hydrogel-grafted fabric. The high-intensity ultrasound (US) prevents the aggregation of NPs resulting in the formation of stable and uniformly deposited ZnO oxide nanoparticles functionalized on hydrogel-grafted silk fibroin fabric dressing.

## 3. Characterization

### 3.1. Surface Morphology

The surface morphology of the hydrogel grafted and un-grafted silk fabric was examined by scanning electron microscopy (SEM) (Carl Zeiss, Oberkochen, Germany) at an accelerating voltage of 15 KV. The samples were gold-sputter coated to render them electrically conductive and fixed by adhesive tape in the sample stage before mounting on the SEM machine. The elemental analysis of the grafted and un-grafted hydrogel silk fibroin was carried out using energy-dispersive X-ray spectroscopy (EDX). Transmission electron microscopy (TEM) (Carl Zeiss, Germany) was used to find the surface morphology of zinc oxide nanoparticles where samples were mounted after making 1mg/mL nanoparticle solution followed by 20 min sonication.

### 3.2. Structural Characterization

The conformation of grafting on silk fabric was carried out by comparing the infrared spectra using Scientific Nicolet 380 spectrophotometer, USA using transmittance mode. Scanning was carried out in the frequency range of 500 cm^−1^ to 2000 cm^−1^ using KBr pellet. KBr pellet was prepared by 1 part of the fine piece of fabric sample with 20 parts of KBr. FTIR spectra were recorded after 64 scans at a resolution of 4 cm^−1^.

### 3.3. Mechanical Properties

Tensile properties of different dressing materials were measured after immersion in water for 24 h as per the protocol reported by Purwar et al. [17]. The samples were cut in form of rectangular strips as per ASTM D 882 (50 mm × 10 mm × 0.4 mm) and analyzed on Instron 3369 (Universal Testing Machine) equipped with 50 kN load cell at a crosshead speed of 5 mm/min. The experiments were performed in triplicate and tensile properties are reported as average ± Standard Deviation.

### 3.4. Hydrophilicity Measurements

#### 3.4.1. Swelling Ability

The swelling ability of the hydrogel coated silk woven fabric was studied at 37 °C as per protocol [18]. The dried sample of the hydrogel coated silk woven fabric (2 cm × 3 cm) were weighed to determine the weight of the dry sample. After weighing, the samples were immersed in distilled water at 37 °C. The percentage of swelling was determined by measuring the weight of dry and wet samples at regular intervals until saturation. The swelling ratio is expressed in Equation (1):

Swelling ratio (%) = [(W_s_ − W_d_)/W_d_] × 100 (1)

where W_s_, W_d_ represents the weight of the swollen and dry sample respectively [18,19,20].

#### 3.4.2. Water Vapor Transmission Rate (WVTR)

Ideal wound dressing should have optimum WVTR. At low WVTR, the water vapor accumulates beneath the wound dressing and high WVTR results in the drying of the wound. Both conditions are highly prone to infection [21]. WVTR of the different samples was determined with the standard procedure described below [22].

The WVTR is the transmission rate of water vapor through a unit area of a body under specific conditions of temperature and humidity. The WVTR was determined gravimetrically using the prime cup method. For this purpose, a plastic cup of a known amount of water was sealed carefully with the hydrogel grafted and un-grafted silk woven fabric using parafilm tape. The initial weight of the beaker and area of the films was measured and kept in the environment of 37 ± 0.5 °C temperature and 40 ± 2% relative humidity for 24 h and then weighed again. The WVTR is expressed in g/m^2^/day.

WVTR = (W_i_ − W_t_)/AT (2)

where Wi = Initial weight of the assembly in grams; Wt = Weight of the assembly in grams after time t; A = WVTR test area of the sample in m^2^, T = Time duration between W_i_ and W_t_ in hours [22,23].

### 3.5. Biocompatibility Test

#### 3.5.1. Cellular Proliferation of L929 Fibroblast Cells

L929 cells were seeded in 24-well tissue culture plates at a density of 40,000 cells per well. The cells were treated with silk fibroin fabrics (SF), hydrogel grafted silk fibroin (SH), and ZnO oxide nanoparticles functionalized on hydrogel-grafted silk fibroin fabric (SH-ZnO) dressing materials (disc shape ~2 cm diameter) at sub-confluence (~70%) for 24 h. The volumetric ratio is defined as the volume of NPs to the volume of Opti-MEM media. After treatment and washing the cells twice with 1× phosphate buffer saline (PBS), the media was replaced with fresh DMEM media. The cells were imaged using live-cell microscopy at 24 h, 72 h, and 120 h respectively. The media was replaced every second day during the experiment.

#### 3.5.2. MTT Assay for Assessing Cytocompatibility of Different Dressings

The L929 cells were seeded at a density of 40,000 cells per well in 500 μL DMEM culture medium with 10% FBS in 24-well-plates, followed by incubation at 37 °C. After 24 h, the cells were treated with SF, SH, and SH-ZnO, the dressing material was cut into a disc shape with ~2 cm diameter before treatment. The dressing material was removed and the cells were washed with 1× PBS, followed by the addition of 1 mL fresh DMEM media to each well. Subsequently, 300 μL of MTT reagent (concentration ~1 mg/mL) was added to the media and the cells were further incubated for 3 h. The purple formazan crystals that formed were dissolved in 100 µL of dimethyl sulfoxide (DMSO). The absorbance was measured using an ELISA reader at a wavelength of 540 nm and a reference wavelength of 620 nm. The percentage of viable cells was expressed considering untreated cells as 100% viable.

#### 3.5.3. Fluorescence Microscopy for Studying the Effect on Cellular Morphology

The effect of SF, SH, and SH-ZnO dressing materials on L929 cellular morphology was studied using a fluorescence-based approach. The L929 cells were seeded at a density of 40,000 cells per well in 24 well plates and allowed to reach sub-confluency (~70%). The cells were then washed twice with 1× PBS and incubated with different dressing materials for another 24 h. Live-cell fluorescence microscopy was performed to observe the change in cellular morphology. The cytoplasm of the murine fibroblast cell line was stained with Calcein-AM (Thermo Fischer Scientific, Waltham, MA, USA, C1430) dye and the nucleus was stained using Hoechst stain (Thermo Fischer Scientific, 33342).

#### 3.5.4. Measurement of Inflammatory Markers

L929 murine fibroblast cells were seeded in 24 well plates at a density of 40,000 cells per well and allowed to grow. These cells were washed twice with 1× PBS and incubated with SF, SH, and SH-ZnO based wound dressing materials (circular shapes of diameter ~2 cm) for another 24 h. The used cell culture medium was collected from each well and subsequently stored at −20 °C after dividing it into small 200 μL aliquots to avoid multiple cycles of thawing. The medium containing extracellular cytokines was thawed just before analysis. Cytokine profiling was done for TNF-α and IL-2 cytokines using commercially available ELISA kits (e-Biosciences, BMS223HS, and BMS238HS, respectively) as per the manufacturer’s protocol.

### 3.6. Antimicrobial Activity

The antibacterial property of SH-ZnO was tested using the agar disc diffusion method according to AATCC 30. The samples were sterilized by UV radiation under laminar airflow, and placed onto the bacterial cultured gel plate prepared by pouring a mixture of Agar-Agar (3% *w*/*v*) and Luria Broth (2% *w*/*v*). The antibacterial activity of ZnO incorporated silk hydrogel was monitored against gram-negative bacterial stain *E. coli* [24]. The plates were inoculated at 37 °C for 24 h in the incubator. After incubation, the size of the zone of inhibition produced around the sample was evaluated.

## 4. Results and Discussion

### 4.1. Synthesis and Structural Characterization

**Scheme 1:**
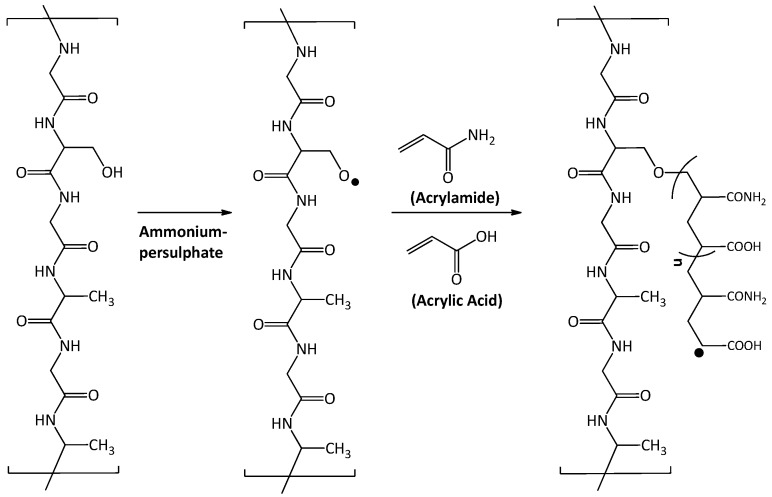
Grafting of silk fabric with poly-acrylic acid-acrylamide.

The grafting of silk with hydrogel was carried out in one pot involving three steps. (i) Activation of silk fibroin by ammonium persulphate (APS), (ii) Reaction of activated silk fibroin with acrylic acid and acrylamide, and (iii) Crosslinking of grafted polymer chains using MBA. The grafting of silk fabric with poly-acrylic acid-acrylamide has been described in Scheme 1. The −OH group present in silk fibroin, on reaction with APS, resulted in the formation of −O free radical which activates the process of polymerization in the presence of acrylic acid and acrylamide monomers. This results in the formation of a covalent bond between the monomer units and silk fibroin (Scheme 1). However, it should be mentioned that other non-covalent interactions such as electrostatic, hydrophobic, and ionic interaction may play minor roles in the attachment of the polymer to the silk substrate.

**Scheme 2:**
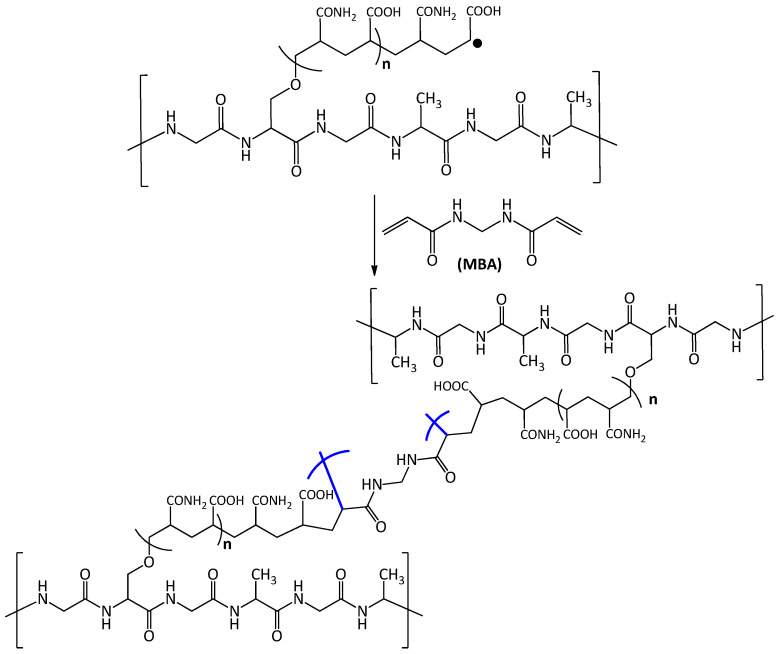
Crosslinking of poly-acrylic acid-acrylamide chains using MBA.

The reaction of silk fabric grafted with poly-acrylic acid-acrylamide with MBA results in the formation of hydrogel like structure due to the crosslinking (Scheme 2).

**Figure 1:**
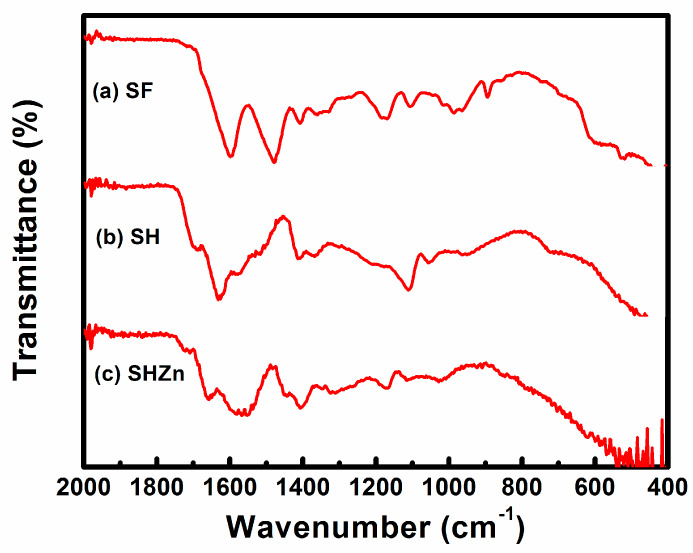
FTIR spectra of (**a**) SF, (**b**) SH, (**c**) SH-ZnO.

**Figure 2:**
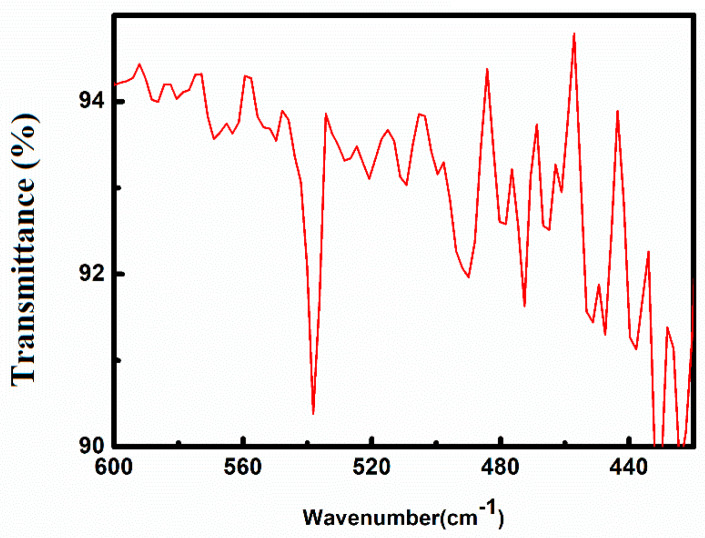
FTIR spectra of ZnO functionalized hydrogel grafted silk dressing.

FTIR analysis has been carried out in order to study the conformation of silk fibroin as well identification of functional groups in the ZnO-functionalized hydrogel-grafted silk dressing. A comparative depiction of FTIR spectra of pure silk (SF), hydrogel-grafted silk (SH), and ZnO-coated hydrogel-grafted silk fabric (SH-ZnO) has been given in Figure 1. The presence of stretching of the amide I, amide II, and amide III region at 1623 cm^−1^, 1512 cm^−1^, and 1229 cm^−1^ respectively, suggest a β sheet structure of pure silk fibroin protein {Figure 1(a)} [25]. The peak at 3338 cm^−1^, 1710 cm^−1^, and 1650 cm^−1^ [17] are attributed to the N-H stretching, C=O of the acidic group, and C=O of acrylamide group respectively (Figure 1b). This confirms the grafting of hydrogel on the silk fibroin fabric. A strong peak at 438 cm^−1^ in the IR spectra of the ZnO-functionalized hydrogel-grafted silk fibroin (Figure 2) is attributed to the stretching vibrations of Zn–O bonds. This finding is in close agreement with the previously reported study [26].

### 4.2. Surface Morphology

**Figure 3:**
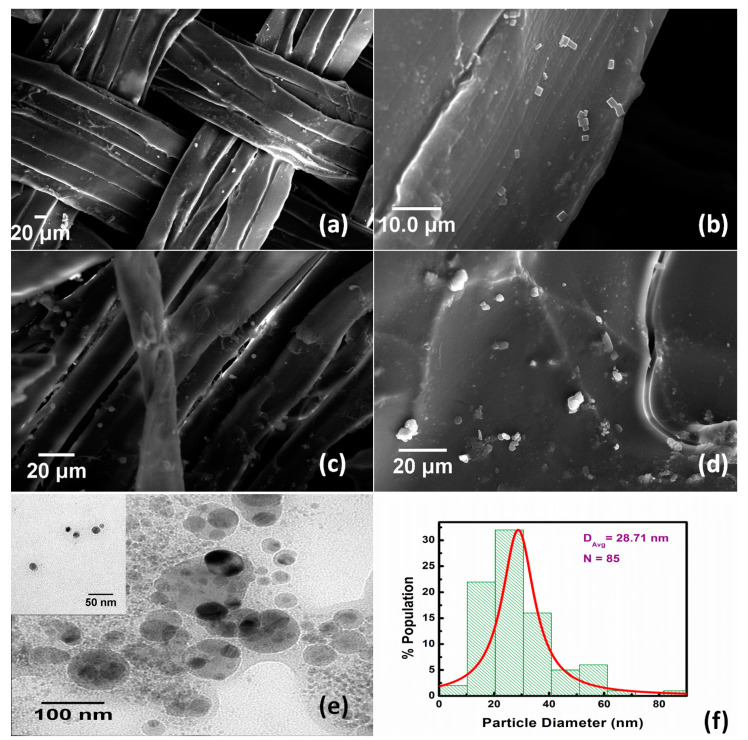
Scanning electron micrographs of ZnO deposited silk woven fabric (**a**), (**b**), ZnO deposited hydrogel coated silk woven fabric (**c**), (**d**). Transmission electrom micrograph of ZnO nanoparticles (**e**), and particle size distribution for ZnO nanoparticles (**f**).

The surface morphology of ZnO coated and uncoated silk hydrogel was examined using SEM (Figure 3). Surface morphology analysis indicated that the ZnO NPs were successfully deposited on the surface of silk fabric (Figure 3a,b) [27]. It is also evident that the hydrogel layer has been deposited on the surface of the fabric, coated with ZnO nanoparticles (Figure 3c,d) [17]. The porosity of the silk fabric slightly reduced after grafting of hydrogel (Figure 3c), which would be beneficial for absorption and retention of exudates. The morphology and microstructure of the ZnO nanoparticles were studied using TEM micrographs (Figure 3e). All nanoparticles were spherical in shape and uniformly dispersed. The particle size of the synthesized ZnO nanoparticles was 28.71 nm (Figure 3f).

**Figure 4:**
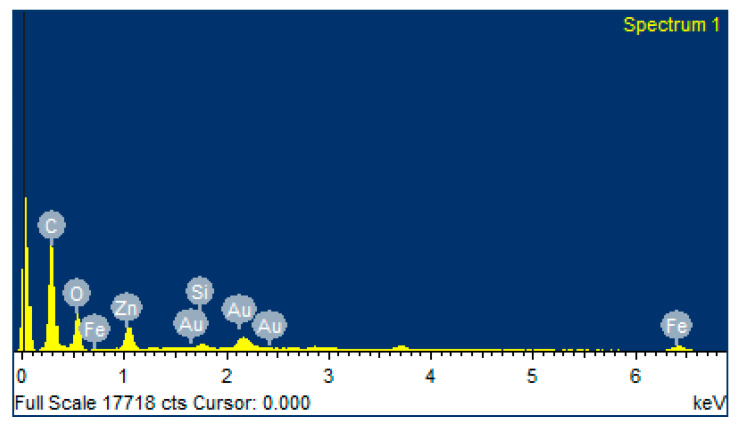
EDX of ZnO functionalized hydrogel grafted silk dressing.

The elemental analysis of zinc oxide nanoparticles functionalized on hydrogel grafted silk fibroin fabrics dressing was carried out using SEM-EDX. It was found that 10.03 wt% of ZnO has been deposited on the surface of hydrogel coated silk woven fabric (Figure 4, Table 1).

### 4.3. Mechanical Properties

The mechanical properties of different dressing samples were measured after immersion in water and the data is shown in Table 2. The tensile strength and modulus of pure tasar fabric film increased after grafting with hydrogel, while its extension at break value decreased. This clearly indicates that the toughness of samples increased after the grafting. Silk fabric acts as a reinforcement to hydrogel in composite dressing. Deposition of ZnO nanoparticles on hydrogel grafted tasar fabric does not show any significant increment in the tensile properties of hydrogel grafted tasar fabric.

### 4.4. Hydrophilicity Measurements

#### 4.4.1. Swelling Ability

**Figure 5:**
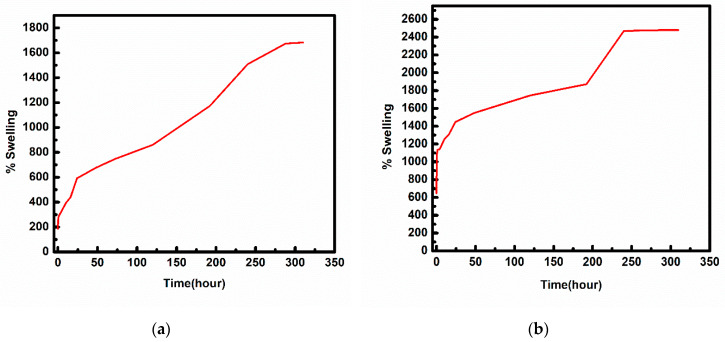
% Swelling of hydrogel grafted silk fabric, (**a**) 5% (**b**) 25% APS.

An ideal wound dressing should absorb a good amount of exudate and provide a moist environment for the wound. Water absorption plays a crucial role in biomaterials used as wound dressings as it prevents the accumulation of exudate which can further cause bacterial infection. The water absorption capacity (swelling) of hydrogel-grafted silk dressing has been optimized as a function of initiator and time as given in Figure 5. The water-uptake capacity has been found to increase with time for each type of sample and the samples were found to be saturated in 3 weeks. The water-uptake capacity of the hydrogel coated silk sample in the presence of 5% and 25% APS has been found to be 1700% and 2500% respectively (Figure 5). The swelling ratio for raw and treated polyester fabric with corn silk/zinc oxide nano-composites displayed 116.7% and 200% [20]. The high swelling property of hydrogel-grafted silk fibroin fabrics is indicative of its hydrogel properties. Thus, the hydrogel-grafted silk fabric could be a better biomaterial to be used as wound dressings.

#### 4.4.2. Water Vapor Transmission Rate (WVTR)

**Figure 6:**
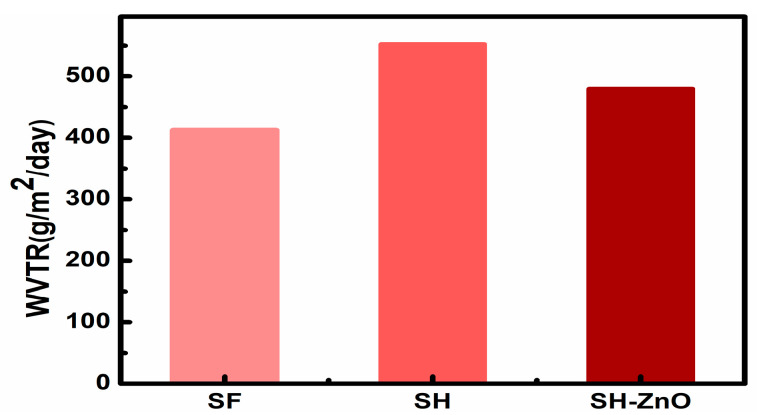
WVTR of different silk samples.

The water-vapor permeability of a wound dressing plays an important role in the prevention of dehydration and inhibits infection at wound sites. A good wound dressing should control evaporative water loss from wounds at an optimal rate. Lamke et al. reported that WVTR for normal and insured skin can range in the order of 204 and 278 g/m^2^/day respectively. The WVTR of different types of wounds and commercially available dressings are summarized in Table 3. Granulating wounds have the highest evaporative water loss (20 times that of normal skin). The water-vapor permeability of wound dressings should prevent both excessive dehydration and the build-up of exudate. The WVTR of hydrogel coated silk was found to be 480 g/m^2^/day which can be suitable for secondary and tertiary wounds (Figure 6).

### 4.5. Cytocompatibility Test

#### 4.5.1. Cytotoxicity Test

**Figure 7:**
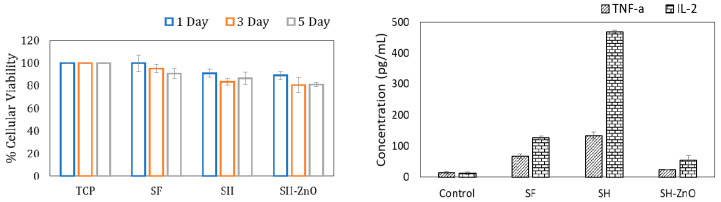
MTT assay and expression of TNF-α and IL-2 by SF, SH, and SH-ZnO.

The dressing material should be porous, biocompatible, and allow the cells to adhere, proliferate, and migrate for effective wound healing. The cytocompatibility of the different dressing materials were tested by measuring cell viability using MTT assay in L929 skin fibroblast cells. The viability of the cells seeded on dressing materials was studied using the MTT assay. The cell-seeded on pure silk fabric shows good viability (Figure 7). The cellular viability of pure silk fabric was found comparable with that of the tissue culture plate (*p* > 0.5). No significant drop in cellular viability was observed in the case of hydrogel-grafted silk dressing with and without ZnO functionalization. MTT-based cytotoxicity testing suggested that the hydrogel and ZnO coating on silk fibroin do not result in significant cellular killing. Immune response profiling in terms of TNF-α and IL2 expression was done with the help of ELISA kits (as mentioned earlier), and the dressing was found to be non-immunogenic.

#### 4.5.2. Phase-Contrast Microscopy

**Figure 8:**
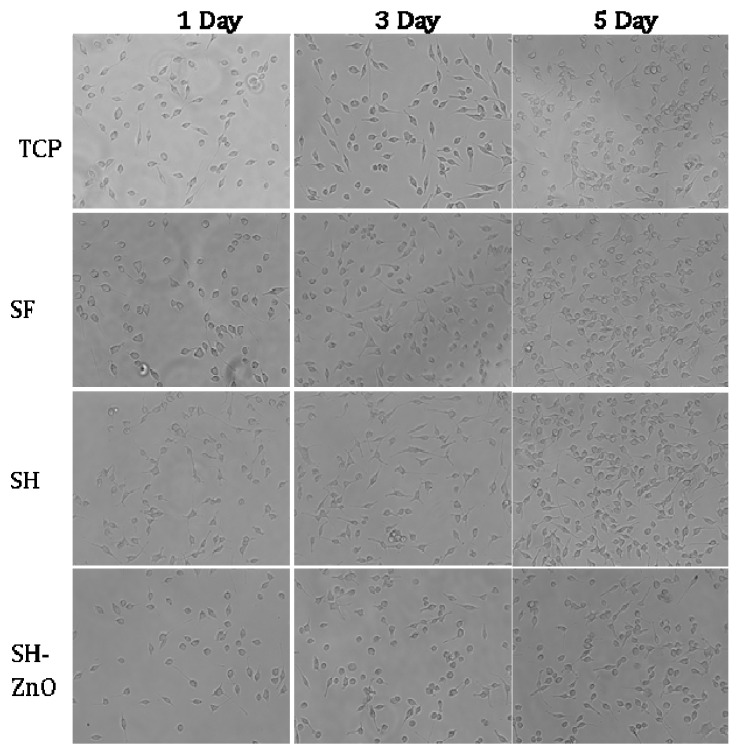
Images of Phase contrast microscopy of different samples.

The morphology and spreading of L929 cells seeded on different samples were studied using phase-contrast microscopy. The cells attained their normal morphology after one day of incubation in each sample (Figure 8). The spreading and proliferation of cells increased with time as compared to the control. There was no significant difference between the density of cells seeded on pure silk and nanoparticles functionalized on hydrogel-grafted silk fabric (*p* > 0.5). The Tasar variety of silk seemed to be ideal candidates for wound dressing applications as it showed a positive effect on cell density and collagen formation at the wound site by enhancing fibroblast growth and proliferation of human skin. This may be due to the presence of tripeptide sequences (R(Arg)-G(Gly)-D(Asp) having positive charge arginine residue. Although, it may be appropriate here to mention the possible partial masking of RGD residues after hydrogel coating. Increased levels of IL-2 helps in the wound healing process whereas TNF-α is considered as a therapeutic target in wound healing. IL-2 expression was significantly higher with SH as compared to the control and PS, whereas the expression of TNF-α by PH and SH was only slightly higher than the control. The expression of TNF-α and IL-2 for ZnO nanoparticles functionalized on hydrogel grafted silk dressing was comparable to the control.

#### 4.5.3. Fluorescence Microscopy for Studying the Effect on Cellular Morphology

Further confirmation of adherence, growth, and proliferation was assessed through fluorescence microscopy after one day of incubation. Fluorescence images of Calcein-stained cells showed a proliferation assay suggesting normal cell density as that of TCP cells. The structural and morphological characteristics of ZnO nanoparticles functionalized on the hydrogel-grafted silk possess the characteristics of natural tissue and indicates that they provide a favorable environment for the exchange of water vapors, water absorption, and metabolite for growth of cells which promote proliferation and adherence of cells on the silk surface [11].

**Figure 9:**
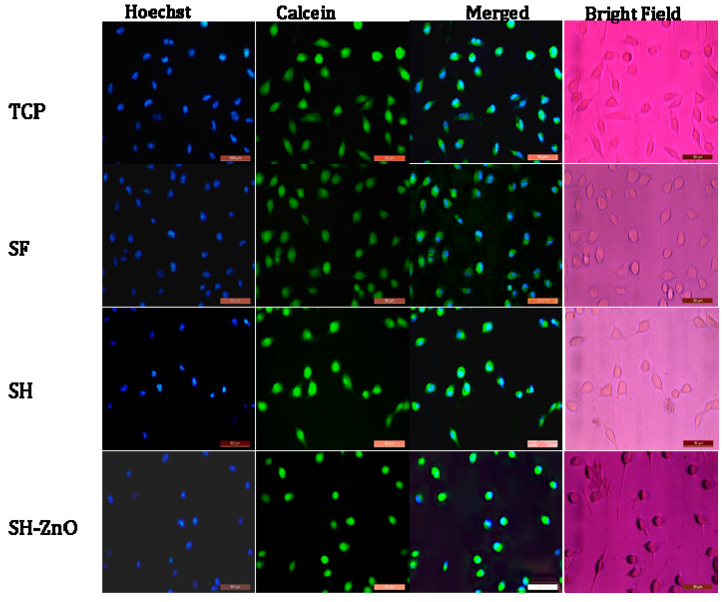
Fluorescence microscopy of cells seeded on different samples after staining with Holchst and Calcein.

Fluorescence microscopic images of Hoechst stained cells (Figure 9) showed the nuclei adhered well to the surface of SF, SH, and SH-ZnO after one day of incubation. The nucleus are round and prominent with an intact cytoplasm. This indicates that the cells adhered well on the dressings and exhibited a normal morphology similar to the control.

### 4.6. Antimicrobial Activity

**Figure 10:**
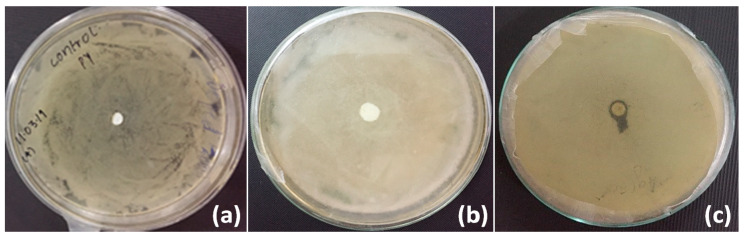
Antimicrobial test for SF as control (**a**), SH (**b**), and zinc oxide coated silk hydrogel (SH-ZnO) (**c**).

The antibacterial activity of ZnO coated silk hydrogel film was analyzed by the agar disc diffusion method using AATCC 30 protocol. The antibacterial activity was monitored against gram-negative bacterial stain *E. coli*. The hydrogel film showed good antibacterial activity in the form of the zone of inhibition (Figure 10). The zone of inhibition was calculated to be 8 mm. SF and SH showed no zone of inhibition.

## 5. Conclusions

A composite wound dressing with a core layer of silk woven fabric and outer layer of poly(acrylic acid-co acrylamide) hydrogel coated with ZnO NPs has been developed. The hydrogel layer was incorporated on the fabric by grafting AAm and AAc monomers on the silk fabric using APS as an initiator and MBA as a crosslinker. Further ZnO NPs were incorporated to obtain ZnO nanoparticles functionalized on hydrogel-grafted silk dressing. Studies suggest that the dressing has sufficient water-vapor permeability, adequate mechanical, and significant antibacterial property. MTT assay and phase contrast microscopic studies showed that the adherence, growth, and proliferation of L929 fibroblast cells seeded on zinc oxide nanoparticles functionalized on hydrogel-grafted silk fibroin fabric dressing was significantly higher than that of pure silk fibroin. These ZnO nanoparticles functionalized on hydrogel-grafted silk dressing could be a promising material for wound dressing and regenerative medicine. In vivo experiments and clinical tests are currently in progress in our laboratory in order to prove the efficacy of the dressing.

## Figures and Tables

**Table 1:** Elemental analysis of zinc oxide coated silk hydrogel dressing.

Element	Weight %	Atomic %
C K	55.32	69.83
O K	28.24	26.76
Si K	0.47	0.25
Fe K	1.91	0.52
Zn K	10.04	2.33
Au M	4.03	0.31
Totals	100	100

**Table 2:** Mechanical properties of different dressing samples.

Sample Name.	Tensile Strength (MPa)	Tensile Modulus (MPa)	Extension at Break (mm)
SF	20.02 ± 0.39 (0.022)	450 ± 4.0 (0.011)	4 ± 0.16 (0.05)
SH	22 ± 0.21 (0.012)	540 ± 3.6 (0.008)	6 ± 0.65(0.133)
SH-ZnO	22.5 ± 1.3 (0.72)	544 ± 5.3 (0.012)	6 ± 0.69 (0.14)

Values given in bracket are Coefficient of Variation.

**Table 3:** WVTR of different types of wound and different commercially available dressings.

S. No.	WVTR (g/m^2^/day)	References
Primary wound	2000	[21]
Secondary wound	4000	[28]
Tertiary wound	3400	[28]
Granulating wound	5000	[21]
Tegaderm	491	[21]
Op-Site	792	[29]
Hydrogel coated silk	480	
