# Erratum: Majumder, S. et al. Zinc Oxide Nanoparticles Functionalized on Hydrogel Grafted Silk Fibroin Fabrics as Efficient Composite Dressing. *Biomolecules* 2020, *10*, 710

**DOI:** 10.3390/biom10081117

**Published:** 2020-07-28

**Authors:** Sudip Majumder, Ujjwal Ranjan Dahiya, Sunny Yadav, Pratibha Sharma, Debashree Ghosh, Gyandshwar K. Rao, Varun Rawat, Gaurav Kumar, Anuj Kumar, Chandra Mohan Srivastava

**Affiliations:** 1 Department of Chemistry, Amity School of Applied Sciences, Amity University Haryana, Gurugram 122413, India; sudip22m@gmail.com (S.M.); sunnyyadavmed121296@gmail.com (S.Y.); pratibha30sharmas@gmail.com (P.S.); dghosh@ggn.amity.edu (D.G.); gkrao@ggn.amity.edu (G.K.R.); vrawat@ggn.amity.edu (V.R.); 2 CSIR-Institute of Genomics and Integrative Biology, New Delhi 110021, India; ujjwal.ranjan@igib.in; 3 Department of Biochemistry, University of Delhi, South Campus, New Delhi 110021, India; gauravkumar747@gmail.com; 4 School of Chemical Engineering, Yeungnam University, Gyeongsan 38541, Korea; 5 Centre for Polymer Technology, Amity School of Applied Sciences, Amity University Haryana, Gurugram 122413, India

The authors wish to make the following correction to this paper [1].

The affiliation of Dr. Anuj Kumar was incorrect in the published paper in *Biomolecules* [1].

Therefore, this information was changed by the Biomolecules Editorial Office from:

School of Chemical Engineering, Yyeongsan 38541, Korea

To:

School of Chemical Engineering, Yeungnam University, Gyeongsan 38541, Korea

We apologize for any inconvenience caused to the readers. The manuscript will be updated, and the original will remain online on the article webpage with a reference to this Erratum.
